# Three-dimensional atomic-scale observation of structural evolution of cathode material in a working all-solid-state battery

**DOI:** 10.1038/s41467-018-05833-x

**Published:** 2018-08-21

**Authors:** Yue Gong, Yuyang Chen, Qinghua Zhang, Fanqi Meng, Jin-An Shi, Xinyu Liu, Xiaozhi Liu, Jienan Zhang, Hao Wang, Jiangyong Wang, Qian Yu, Ze Zhang, Qiang Xu, Ruijuan Xiao, Yong-Sheng Hu, Lin Gu, Hong Li, Xuejie Huang, Liquan Chen

**Affiliations:** 1 Beijing National Laboratory for Condensed Matter Physics, Institute of Physics, Chinese Academy of Sciences, 100190 Beijing, China; 2 School of Physical Sciences, University of Chinese Academy of Sciences, 100049 Beijing, China; 3 Department of Physics, Shantou University, Shantou, 515063 Guangdong, China; 4 Center of Electron Microscopy and State Key Laboratory of Silicon Materials, School of Materials Science and Engineering, Zhejiang University, 310027 Hangzhou, China; 5 DENSsolutions, Informaticalaan 12, 2628ZD Delft, The Netherlands; 6 Collaborative Innovation Center of Quantum Matter, 100084 Beijing, China

## Abstract

Most technologically important electrode materials for lithium-ion batteries are essentially lithium ions plus a transition-metal oxide framework. However, their atomic and electronic structure evolution during electrochemical cycling remains poorly understood. Here we report the in situ observation of the three-dimensional structural evolution of the transition-metal oxide framework in an all-solid-state battery. The in situ studies LiNi_0.5_Mn_1.5_O_4_ from various zone axes reveal the evolution of both atomic and electronic structures during delithiation, which is found due to the migration of oxygen and transition-metal ions. Ordered to disordered structural transition proceeds along the <100>, <110>, <111> directions and inhomogeneous structural evolution along the <112> direction. Uneven extraction of lithium ions leads to localized migration of transition-metal ions and formation of antiphase boundaries. Dislocations facilitate transition-metal ions migration as well. Theoretical calculations suggest that doping of lower valence-state cations effectively stabilize the structure during delithiation and inhibit the formation of boundaries.

## Abstract (web summary)

Here, with the state-of-the-state electron microscope, the authors report three-dimensional atomic-scale observation of LiNi_0.5_Mn_1.5_O_4_ from various directions, revealing unprecedented insight into the evolution of both atomic and electronic structures during delithiation.

## Introduction

LiNi_0.5_Mn_1.5_O_4_ (LNMO) is one of the most promising cathode materials for lithium-ion batteries because of its high voltage, low cost, and non-toxicity, specifically as the demand for solid electrolytes (to replace liquid electrolytes) is increasing due to the safety issues and pursuit of higher energy density in the fields including power electric vehicles (EVs) and smart electric grid^1–6^. While solid electrolytes have many benefits, there are also drawbacks with respect to battery performance including low ion-conductivity, poor contact between electrode and electrolyte, and grain boundary formation^7^. Besides, the high-voltage LNMO cathode suffers severe problems such as dissolution of Mn ions and capacity degradation at high voltage, which hinders its commercialization^8,9^. As an important issue, the structural evolution during charging in LNMO has been broadly studied since it is directly responsible for the performance of batteries. By measuring charge–discharge curves and using X-ray diffraction (XRD), transmission-electron microscopy (TEM), and neutron diffraction, phase-transitions were reported, and two-phase mechanism, three-phase mechanism, and a solid solution were proposed^10–14^. However, understanding the structural evolution and the related intrinsic mechanisms is still lacking due to the technical difficulty of giving direct observation on the dynamic evolution of atomic structure and electronic structure during charge and discharge cycles.

In situ TEM technique is a powerful tool for this study that enables direct observation on the change of microstructure as a certain external field is applied^15–21^. Specifically by combining focused ion-beam (FIB) milling and a chip-based in situ TEM holder, in situ TEM electrochemical observation is achieved with atomic resolution^22,23^. The extraction of lithium ions and the migration of transition-metal ions can be directly observed^24–26^ For example, Tang et al. found that transition-metal ions of LiMn_2_O_4_ move into 16c sites at a surface within a depth of 4 nm^27^. Lin et al. found that subsurface regions of the cycled particles permit migration of transition-metal ions to the empty octahedral sites, where rock salt-like structures form^8^. In addition, the performance of battery was found to depend on the exposed surface^28,29^. While the previous TEM studies mainly focused on <110> zone axis^8,9,27,30–32^, the movement of ions in real three-dimensional lattice remains unknown.

In this work, we investigate the changes of the atomic and electronic structure of spinel P4_3_32 LNMO during delithiation with special attention paid to the behavior of oxygen and transition-metal ions. Importantly the structural evolution of <100>, <110>, <111>, and <112> zone axes is studied to enable a profound understanding in three dimensions. In addition, FIB prepared sample allow us to lift-out the bulk structure of LNMO whereas previous TEM studies mainly focused on surface structure. Along <100>, <110>, and <111> zone axes, we find that the original Ni/Mn ordered structure became totally disordered. In contrast, we find inhomogeneous structural evolution along <112> zone axis during which Ni ions are more likely to occupy the 4a (16c in Fd–3 m space group) sites than Mn ions. A new evolution process is revealed that is significantly different from the previous results from <110> and <111> zone axes. Combining all, we achieve a three-dimensional view of the dynamic atomic structure evolution in LNMO. We believe that the uneven extraction of lithium ions leads to localized migrations of transition-metal ions and formation of antiphase boundaries. The existence of dislocations facilitates the migration of transition-metal ions as well. Because of the uneven solid–solid interface between cathode and electrolyte, boundaries, and distinct regions form during the evolution of the electronic structure, which may have significant influence on battery performance.

## Results

### Construction of the micro-battery and its pristine structure

**Fig. 1:**
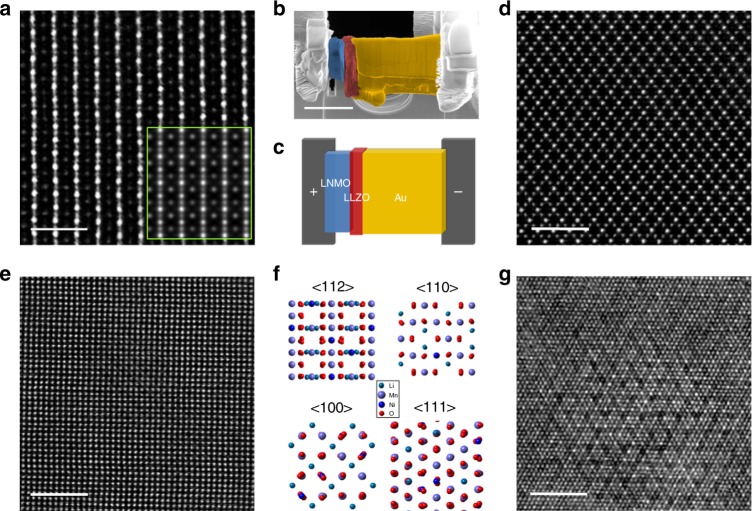
Configuration of the assembled all-solid-state lithium-ion battery and the atomic structure of LNMO of four different zone axes. **a** Atomic scale HAADF-STEM of the pristine LNMO cathode along the <112> zone axis. The simulation HAADF-STEM image is shown in a green rectangle which agrees perfectly with the experiment result. **b** SEM image of the assembled all-solid-state lithium-ion battery, and its corresponding schematic (**c**). **d**, **e**, **g** Atomic scale HAADF-STEM images of LNMO of the <110>, <100>, and <111> zone axes. **f** Structure models of LNMO along <112>, <110>, <100>, and <111> zone axes. Scale bar, 1 nm (**a**), 5 μm (**b**), 2 nm (**d**, **e**, **g**)

We use FIB milling to prepare the all-solid-state lithium-ion battery for in situ STEM observation. The combination of a chip-based in situ biasing holder and an aberration-corrected sub-Å resolution transmission-electron microscope enables a direct observation on the atomic and electronic structural evolution. The detailed parameters and procedures of the FIB milling process, battery assembling, and the in situ setup can be found in a previously published paper by our group and also in Supplementary Figure 1^22^. Fig. 1b, c show SEM images of the assembled all-solid-state lithium-ion battery on the chip and the corresponding schematic. Figure 1a, d, e, and g show pristine atomic scale high-angle annular dark-field scanning transmission-electron microscopy (HAADF-STEM) images of spinel LNMO along the <112>, <110>, <100>, and <111> zone axes. The inset with the green frame in Fig. 1a represents the corresponding simulation HAADF-STEM image of the LNMO along the <112> zone axis. Results of the simulation and the structural data are consistent with the experiment results. Figure 1f illustrates the structure models of <112>, <110>, <100>, and <111> zone axes. Supplementary Figure 2 and 3 show the low magnification STEM images of FIB fabricated sample and scanning electron microscopy (SEM) image illustrating full-cell configuration. We also carried out XRD and SEM measurements on the pristine LNMO particles (see Supplementary Figure 4), which reveals the macro-scale structure and the morphology of the cathode material we used. The in situ TEM tests were carried out in an aberration-corrected microscope with an electric field applied on the chip (and the sample) by manually tuning the voltage of the power source with step size of 0.1 V. Detailed information of the charging condition is demonstrated in Supplementary Figure 5.

### Atomic scale structural evolution of LNMO after delithiation

**Fig. 2:**
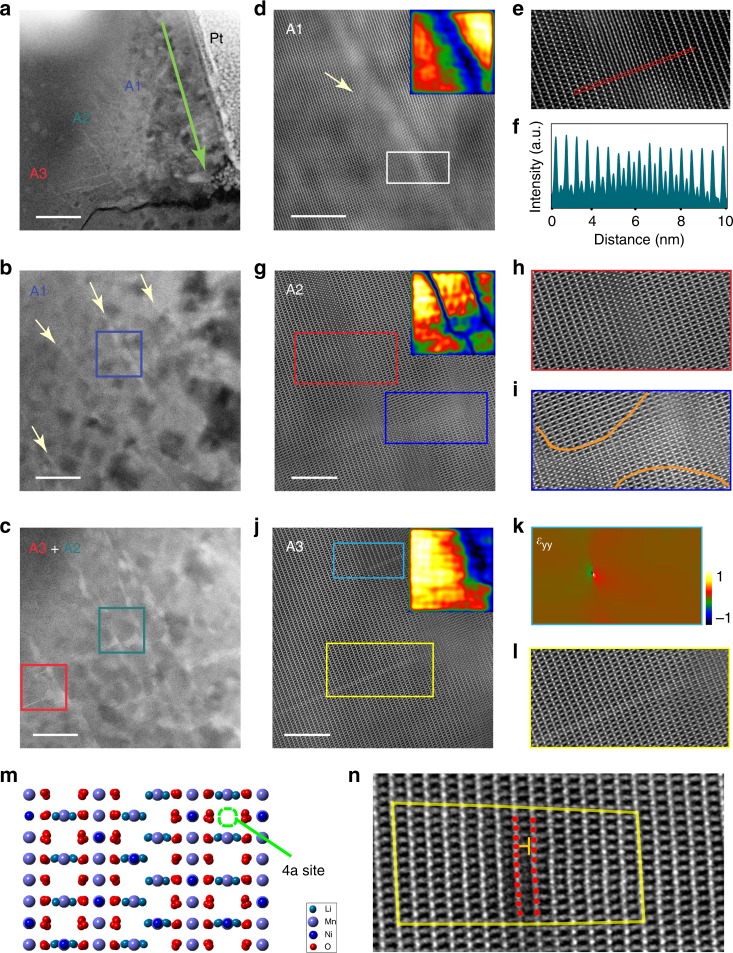
Structural evolution of LNMO after delithiation. **a** Low magnification HAADF-STEM image with three regions marked with A1, A2, and A3. Right side of the image is the Pt deposition on the LNMO. The green arrow indicates the direction of the electric field of all STEM images in Fig. 2. **b** Zoomed-in HAADF-STEM image showing the features of A1 region. Beige arrows show the position of transition-metal-rich areas. **c** Zoomed-in HAADF-STEM image showing the features of A2 and A3 regions. Scale bars of (**a**, **b**, **c**) are 100 nm, 20 nm, and 20 nm, respectively. **d** Atomic scale HAADF-STEM image of A1 region. Inset is the GPA scale image, which can clearly show feature of A1. **e** Enlarged image of the white rectangle in **d**. **f** Intensity line profile extracted from the red rectangle in **e**. It clearly shows the presence of transition-metal ions at the 4a sites. **g** Atomic scale HAADF-STEM image of A2 region and the inset is its corresponding GPA scale image. **h** Enlarged image of the red rectangle in **g**, which shows the structure of the antiphase boundary. **i** Enlarged image of the blue rectangle in **g**, which shows the structure of linking area between two antiphase boundaries. **j** Atomic scale HAADF-STEM image of A3 region and its corresponding GPA scale image at the top right. **k** GPA ε_yy_ image of the light-blue rectangle in **j**, which clearly shows the presence of a dislocation. **l** Enlarged image of the yellow rectangle in (**j**). **m** Atomic structure model of antiphase boundary with marked 4a site. **n** Enlarged HAADF-STEM image of light blue rectangle in **j**, which shows the structure of an edge type partial dislocation. Scale bars of **d**, **g**, **j** are all 5 nm

After delithiation, three different regions appeared along <112> zone axis. They are marked as A1, A2, and A3 in Fig. 2a according to different structures and different locations. The direction of the electric field and the Pt protection deposition are indicated by the arrow and text in Fig. 2a, respectively. The green arrow in Fig. 2a represents the direction of electric field for all STEM images in Fig. 2. Figure 2b, c are low magnification HAADF-STEM images of A1, A2, and A3 regions. The atomic structure of these three areas is shown in the high magnification HAADF-STEM images in Fig. 2d–l.

The regions from A1 to A3 are in successive order (from right to left)—see Fig. 2a. A1 is the region with intact crystal structure appeared as a 3–4 nanometers wide area with bright contrast. A2 is the central region, which contains antiphase boundaries aligned with the direction of the electric field; the bright contrast boundaries perpendicular to the electric field link every two antiphase boundaries. A3 is the forefront area, which only contains bright-contrast boundaries perpendicular to the electric field and connects with the antiphase domain-boundaries at the back.

**Fig. 3:**
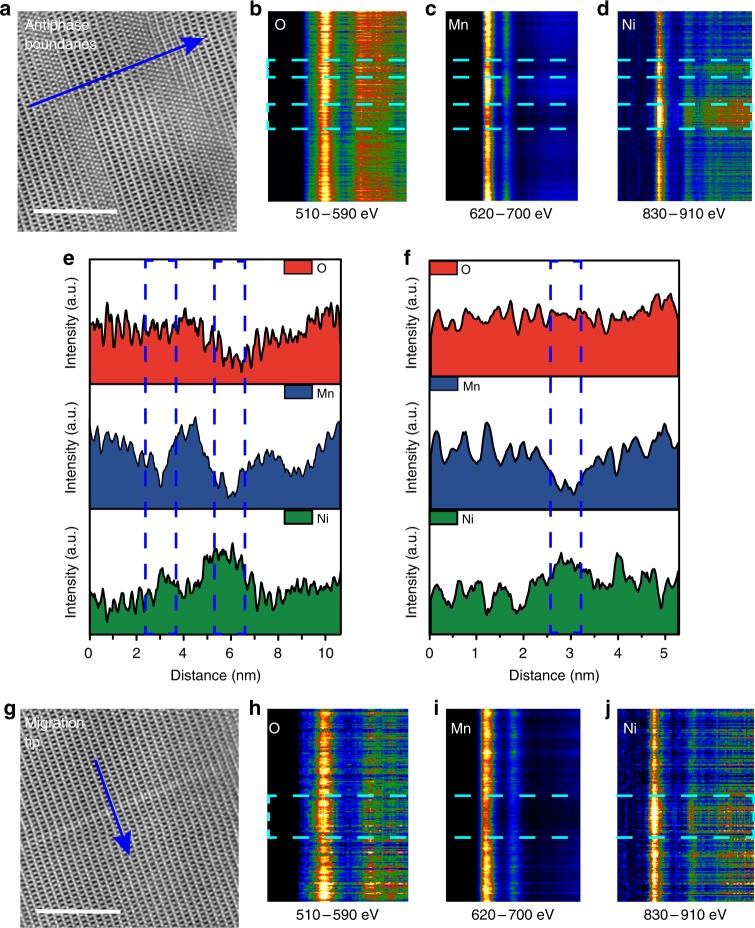
Electronic structural evolution of antiphase boundaries and transition-metal ion migration front after delithiation. **a** Atomic scale HAADF-STEM image of antiphase boundaries with blue arrow showing the EELS line scan trajectory. **b**–**d** EELS spectrum of O, Mn, and Ni extracted from the line scan result of **a**. Light blue dashed line rectangle indicates the position of two antiphase boundaries in **a**. We can see the Mn edge shifts to left (lower energy) and Ni edge shifts to right (higher energy), which means the valence state of Mn decreased and Ni increased. **e** Element content results of EELS line scan of **a**, showing the content fluctuation of O, Mn, and Ni. The results show the O and Mn content decreased, Ni content increased at antiphase domain boundaries. **g** Atomic-scale structure of the migration-front area with a blue arrow indicates the EELS line scan trajectory. **h**, **i**, **j** EELS spectrum of O, Mn, and Ni from **g**. Light blue dash line rectangle indicates the position of the migration front in **g**. Due to the edge shift of Ni and Mn, the valence state of Mn decreased and Ni increased. **f** Element content results of EELS line scan of **g** shows that the Mn content decreased, while the Ni content increased, and O content remains mostly constant at the migration front. Scale bar, 5 nm

Figure 2d shows a high-magnification HAADF-STEM image of area A1. The boundaries are apparently brighter in our Z-contrast HAADF-STEM images since the 4a sites are occupied by heavy elements. Combined with the geographic phase analysis (GPA) at the top right in Fig. 3a, we concluded that these are transition-metal ions that occupy the 4a sites. Through intensity line profile we clearly see that there is a Gaussian like 4a site occupation distribution from the line region center to the edges in area A1.

In area A2, we see antiphase boundaries and a bright area perpendicular to the electric field, which connects every two antiphase boundaries. From the high magnification HAADF-STEM image in Fig. 2g, it is observed that the antiphase boundaries in A2 are different from the common type. Here, the boundary is formed by losing one transition-metal-rich layer along the (111) crystal plane; there is a half unit-cell length mismatch along the (111) crystal plane between the two parts divided by the boundary. The structure model of this type of antiphase boundary is demonstrated in Fig. 2m and Supplementary Figure 6. The distribution of the antiphase boundaries becomes clearly visible in GPA scale image (see inset of Fig. 2g). The linking area with the bright contrast is similar to the A1 region where transition-metal ions occupying 4a sites.

The A3 region is the forefront part of all these three regions. We can see bright single lines (Fig. 2j) emerging perpendicularly from the antiphase boundary. In the magnified image, this line is bright because the 4a sites are occupied by the transition-metal ions. Using a GPA ε_yy_ image, we clearly locate a dislocation near the tip at the front. In the magnified HAADF-STEM image in Fig. 2n, we observed an edge type partial dislocation with Burgers vector of 1/2 <–110>. The presence of both the dislocation and the linking areas between two antiphase boundaries explained the formation of both the antiphase boundaries and the migration front.

### Atomic scale electronic structural evolution in LNMO after delithiation

The electronic structure of the antiphase boundary and migration front are studied. Figure 3a shows the HAADF-STEM image of the antiphase boundaries and the probe-scan trajectory (blue arrow in Fig. 3a) of electron energy-loss spectra (EELS). Figure 3b–d show EELS spectra of O, Mn, and Ni. We found that the L-edge of Mn shift to the left, and the L-edge of Ni shift to the right. This indicates a decrease of the valence state of Mn and an increase of the valence state of Ni. We also find evidence for a fluctuation in the concentration of each transition metal via EELS spectrum. The content distribution of O, Mn, and Ni along the arrow in Fig. 3a is shown in Fig. 3e, which demonstrated the loss of O and Mn, and the enrichment of Ni at antiphase boundaries.

Figure 3f–j show EELS data of the migration-front region. We obtained almost the same results with respect to antiphase boundaries. A loss of Mn and enrichment of Ni are evident at the migration-front area as shown in Fig. 3f. However there is no obvious change in oxygen content. EELS spectra in Fig. 3h–j show the shift of the L-edge of Ni and Mn in the migration area, indicating a decrease of the valence state of Mn and an increase of the valence state of Ni. Overall, Ni is more likely to occupy the 4a site than Mn during delithiation along with the electronic structural evolution at the same time.

### Atomic scale structural evolution along <111>, <110>, and <100> zone axes

**Fig. 4:**
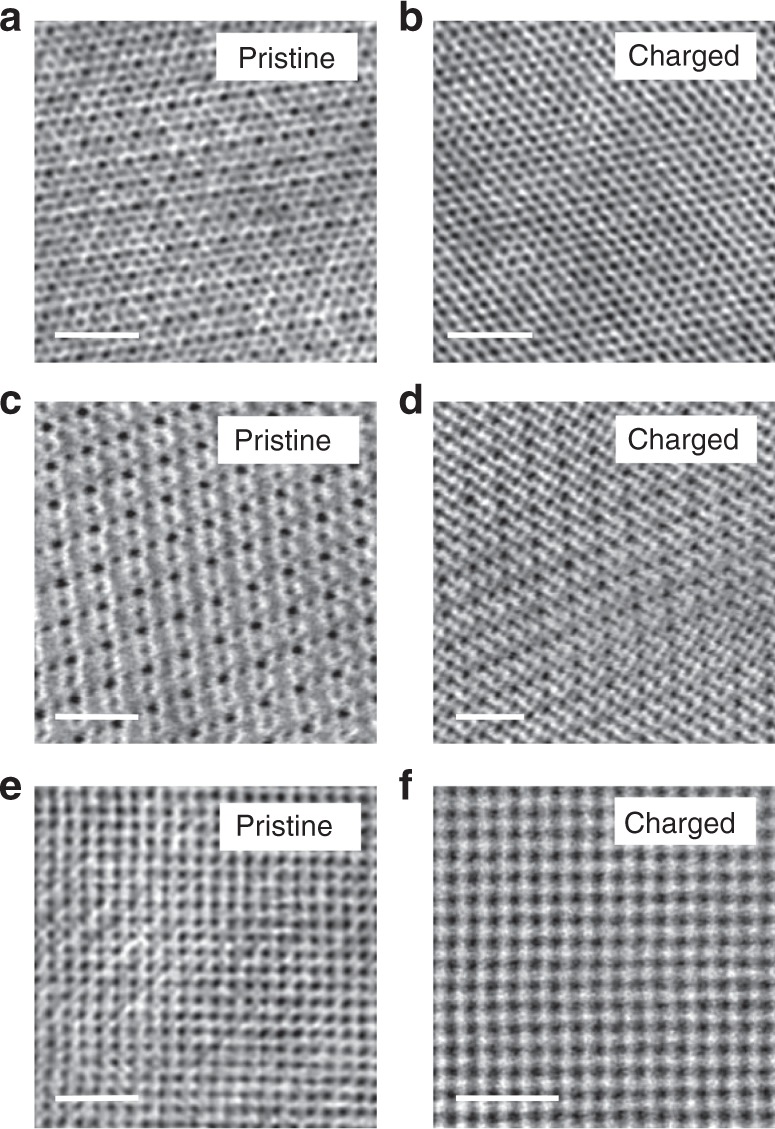
Atomic scale structural evolution of <111>, <110>, and <100> zone axes. **a**–**f** are ABF-STEM images of pristine and charged LNMO along <111>, <110>, and <100> zone axes. All results indicate the transformation from ordered structure to disordered structure, which is different from the inhomogeneous results of <112> zone axis. Scale bars, 1 nm

To understand the structure and electronic evolution in three dimensions, we also performed in situ study along <111>, <110>, and <100> zone axes. Figure 4 shows the annular bright field (ABF)-STEM image of delithiated LNMO, which reveals the evolution from ordered to disordered structure. In Fig. 4c, d, it is found that the transition metal occupies 4a sites after charging. These results are consistent with those of the <112> zone axis and other previous ex situ studies (Supplementary Figure 7). Importantly, beyond the previous understanding which demonstrated that the 4a sites at the surface could be occupied, the result of the <112> zone axis shows that the transition-metal ion migration is not homogeneous while the projection of antiphase boundary structure in the <112> zone axis makes it appear homogeneous along the <111> and <110> zone axes. EELS spectra of these three zone axes is demonstrated in Supplementary Figure 8 and 9, indicating homogeneous electronic structure distribution.

## Discussion

**Fig. 5:**
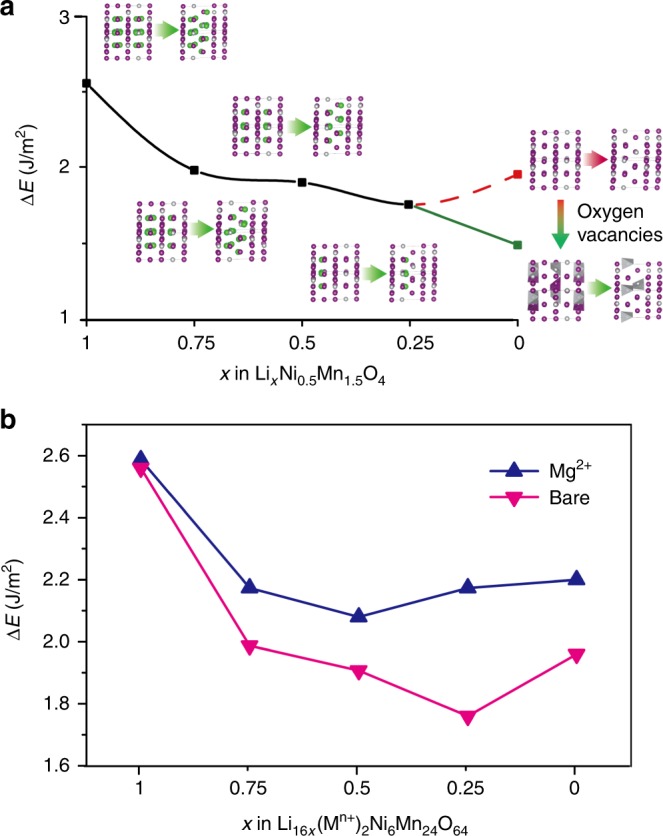
Theoretical calculation result of the interphase energy and the extraction of lithium ions. **a** Density functional theory (DFT)-based first-principles calculations of the energy difference between antiphase boundary and bulk. The structure models for the calculations are shown at each point. The red dashed line indicates the result without considering oxygen vacancies. The green line shows the result considering oxygen vacancies at a high delithiation state. **b** Theoretical calculation results of the interface energy. The pink line shows the result of the bare structure. The blue color curve is from the Mg^2+^ doping setup

To help understanding the experimental results, density functional theory (DFT)-based first-principles calculations was further applied to study the behavior of the transition-metal ions and the formation of antiphase boundaries during delithiation. Both primary and reconstruction supercells with five different Li concentrations *x* (*x* in Li_*x*_Ni_0.5_Mn_1.5_O_4_, *x* = 1, 0.75, 0.5, 0.25, and 0) were constructed to evaluate defect formation energies—see Fig. 5. For pristine (*x* = 1) supercells, a formation energy of 2.56 J m^–2^ is required as the primary structure transforms to a reconstructed structure. During delithiation process, as the decrease of Li content from *x* = 1 to *x* = 0.25, the energy gap continuously decreased to 1.76 J m^–2^. It suggests that extraction of Li tends to diminish the energy discrepancy between the reconstructed and primary phases, making the reconstructed phase energetically competitive.

However, the energy gap increased to 1.96 J m^–2^ when Li content x is below 0.25. Moreover, the concentration of transition-metal ion is low in the dislocation layers in the reconstructed supercell. Excessive Li extraction simultaneously leads to a local decrease of Ni, Mn, and Li ions’ concentration. Consequently, oxygen escapes from the unstable frame. Since stoichiometric oxygen calculation models are not suitable for delithiated conditions, we fabricated the oxygen deficient delithiated supercells by removing two oxygen atoms from both the reconstructed and the primary phases. Due to oxygen vacancies, the defect formation energy was reduced to 1.49 J m^−2^, suggesting that delithiation and oxygen vacancies are two synergetic factors that promote defect formation (see calculation results in Fig. 5a).

By combining in situ STEM results and theoretical calculations, the dynamic picture of the atomic and electronic structural evolution of LNMO cathode during delithiation is constructed. In liquid electrolyte lithium-ion batteries, transition-metal ions dissolve into electrolyte. However, in solid electrolyte lithium-ion batteries, transition-metal ions are more confined within the solid electrolyte. At the beginning, lithium ions were extracted due to the electrochemical force. However, the contact condition between electrode and solid electrolyte are not constant. Because of the compatibility and complexity between and within electrode and solid electrolyte, lithium ions leave the LNMO lattice with different speeds^33,34^, resulting in the formation of different zones with different delithiation levels along the electric field. It has been shown that transition-metal ions would migrate into the 4a (16c in Fd-3m space group) sites of spinel LNMO during the battery cycle. As a result, an area with high-level delithiation will have more transition-metal ions migration than areas with lower-level delithiation. Here, the high-level delithiation zone represents the bright line area of the A1 region in our results. In addition, practical defects exist in LNMO cathode too as shown in the feature at the top of migration front region in Fig. 2j. Together with the extraction of lithium ions, lattice parameters change, which introduces strain into the LNMO crystal. The energy hidden in the dislocation tends to be released to reduce strain and then antiphase boundaries are formed. Furthermore, dislocations can absorb transition-metal ions to dislocation core. It could also explain the origin of 4a sites’ transition metal at the migration front (A3 region) and in the linking area of the A2 region. However, the formation sequence of antiphase boundary and the migration front is hard to determine. Presumably with the help of dislocations and uneven level of delithiation, transition-metal ions can migrate along a particular path.

As also suggested by previous studies, the migration of transition-metal, oxygen ions and the existence of antiphase boundaries in the cathode severely affect the electrochemical properties such as cycle ability, rate ability, and battery capacity^8,9^. Atomic scale elemental doping of Ni and/or Mn in LNMO has been widely accepted as a strategy to control the crystallographic properties and stabilize the battery performance of the high-voltage spinel cathodes^35^. In this study, by applying theoretical calculation, it is found that doping low valence-state cations would hinder the formation of antiphase boundaries and enhance the structural stability. Different valence-state cations were tested. M-doped supercells, i.e., Li_16×_Ni_6_M_2_Mn_24_O_64_ (*x* = 1, 0.75, 0.5, 0.25, or 0, *M* = Ti^4+^, Al^3+^, or Mg^2+^), were built by replacing two Ni ions with two M ions in the un-doped supercells—as mentioned above. For the doped primary supercells, M ions were used to obtain the minimum energies. For the doped reconstructed supercells, M ions were placed in the interchanged layers. Constructed models for the calculations and results of all cations are shown in Supplementary Table 1 and Supplementary Figure 10 and 11. Interface energy curves of LNMO with different doping cations were compared as shown in Fig. 5b and Supplementary Figure 12. Mg^2+^ doping increases the interface energy for all Li contents. Ti^4+^ doping, in contrast, slightly decreases the interface energy, and Al^3+^ shows no significant effect. Moreover, the reconstruction process leads to excessive O^2−^ in the reconstruction region, where cations are easily oxidized to a higher valence state. Ti^4+^ and Al^3+^ show high valence states compared to Mg^2+^. Thus, they do not reject the reconstruction process. In contrast, the appearance of low valence-state cations makes this region energetically unfavorable, indicating that low valence-state dopants such as Mg^2+^ may effectively impede the structural reconstruction.

In conclusion, we used state-of-the-art atomic scale in situ STEM methodology and theoretical calculations to visualize the delithiation of a single-crystal LNMO cathode in three dimensions. Both in situ results of the <100>, <110>, <111>, and <112> zone axes show transition-metal ions migrating to the 4a (16c in Fd-3m space group) sites of the LNMO cathode. Ordered to disordered structural evolution was observed along <100>, <110>, and <111> zone axes and inhomogeneous structural evolution after delithiation was observed along <112> zone axis. Under <112> zone axis, three different regions (Transition-metal-rich regions; Antiphase boundary regions; and Transition-metal migration-front regions) formed during delithiation. Using spectral analysis, we found that Ni ions tend to occupy the 4a (16c in Fd-3m space group) sites than Mn ions, and there are change of valence-state of Ni and Mn with oxygen loss during delithiation. Moreover, our calculations indicate that atomic-scale doping with low valence-state cations can effectively enhance the stability of the LNMO structure. This study not only shows the importance of atomic scale three-dimensional characterizations for improving our understanding on the dynamic process and fundamental mechanisms of delithiation but also sheds light on optimization of the structural stability, as well as the cycle-ability of all-solid-state battery. It may also stimulate atomic scale in situ TEM studies on other promising compounds.

## Methods

### Sample preparation

An all-solid-state lithium-ion battery on the in situ MEMS chip was fabricated using FIB milling. We lifted-out a gold anode, using a LLZO solid electrolyte and LNMO cathode successively. We then connected them with the gold wire in the MEMS chip to form a battery. A set of optimized FIB milling procedures and beam conditions was used to minimize ion-beam damage and assemble the battery efficiently. These procedures and conditions are detailed in our previous publication^22^.

### in situ STEM observations

The all-solid-state lithium-ion battery was prepared on the MEMS device based in situ holder, DENSsolutions Lightning D9+, in a FIB chamber, FEI Helios 6i. STEM images and acquisitions were performed on a probe-aberration-corrected microscope, JEOL JEM-ARM200CF, at 200 kV with 25 mrad incident beam and a collection angle between 90 and 370 mrad for HAADF acquisition, and 11 to 23 mrad for ABF acquisition. STEM-EELS recordings were performed with a Gatan Imaging Filter Quantum Model 965 spectrometer at 200 kV. All spectra were acquired with dwell time as short as 0.05 s of each pixel in line-scan mode. Strain analysis was performed on the basis of the geometric phase analysis method^36^ using FRWR tools plugin (www.physics.hu-berlin.de/en/sem/software/software_frwrtools) for Digital Micrograph. Detailed information can be found in Supplementary Figure 13.

### Theoretical calculations

Spin-polarized calculations were performed using the Vienna Ab-initio Simulation Package (VASP) within the projector augmented-wave approach^37–39^. The generalized gradient approximation (GGA) in the parameterization of Perdew, Burke, and Ernzerhof (PBE) pseudopotential was used to describe the exchange-correlation potential^40^. A Hubbard-type correction U was taken into account due to the strongly correlated nature of the transition-metal 3d electrons^41,42^. According to the literature, the effective U value of Ni, Mn, and Ti ions were set to 6.0, 5.0, and 4.2 eV, respectively^43,44^. The plane-wave cutoff was set to 520 eV. The reciprocal space k-point mesh interval was about 0.03 Å^–1^. Geometry optimizations were performed using a conjugate gradient minimization until all forces acting on ions were less than 0.01 eV/Å per atom. Un-doped supercells, consisting of sixteen Li_x_Ni_0.5_Mn_1.5_O_4_ (*x* = 1, 0.75, 0.5, 0.25, or 0) formula units, were used. The primary supercells were built from bulk spinel structures, while the reconstructed supercells were built by the interchange between two transition-metal layers in the primary supercells.

## Electronic supplementary material

Supplementary Information

## Data Availability

All data supporting this study and its findings are available within the article, its Supplementary Information. Any source data deemed relevant are available from the corresponding author upon request
