# Supplementary material for: Three-dimensional atomic-scale observation of structural evolution of cathode material in a working all-solid-state battery
Source: Nat Commun. 2018 Aug 21;9:3341. doi: 10.1038/s41467-018-05833-x (PMC6104093; doi:10.1038/s41467-018-05833-x)
Supplement: Supplementary file 1 — Supplementary Information [file 41467_2018_5833_MOESM1_ESM.pdf]

Supplementary Information

Supplementary Figures

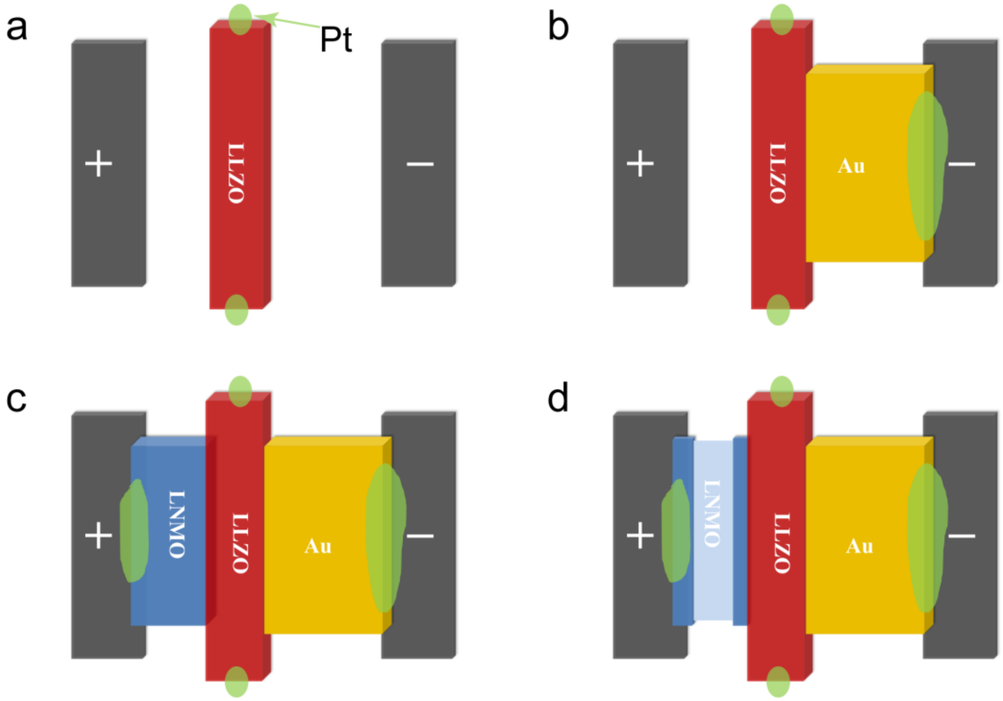

Supplementary Figure 1. Upgraded FIB milling process of the battery assembling. (a) We first use Pt deposition to fix LLZO. (b) Then, we connect Au with LLZO using Pt deposition only on wire side. (c) Next, we connect LNMO cathode with LLZO using Pt deposition only on wire side too. (d) Last, LNMO cathode is thinned carefully for STEM observation.

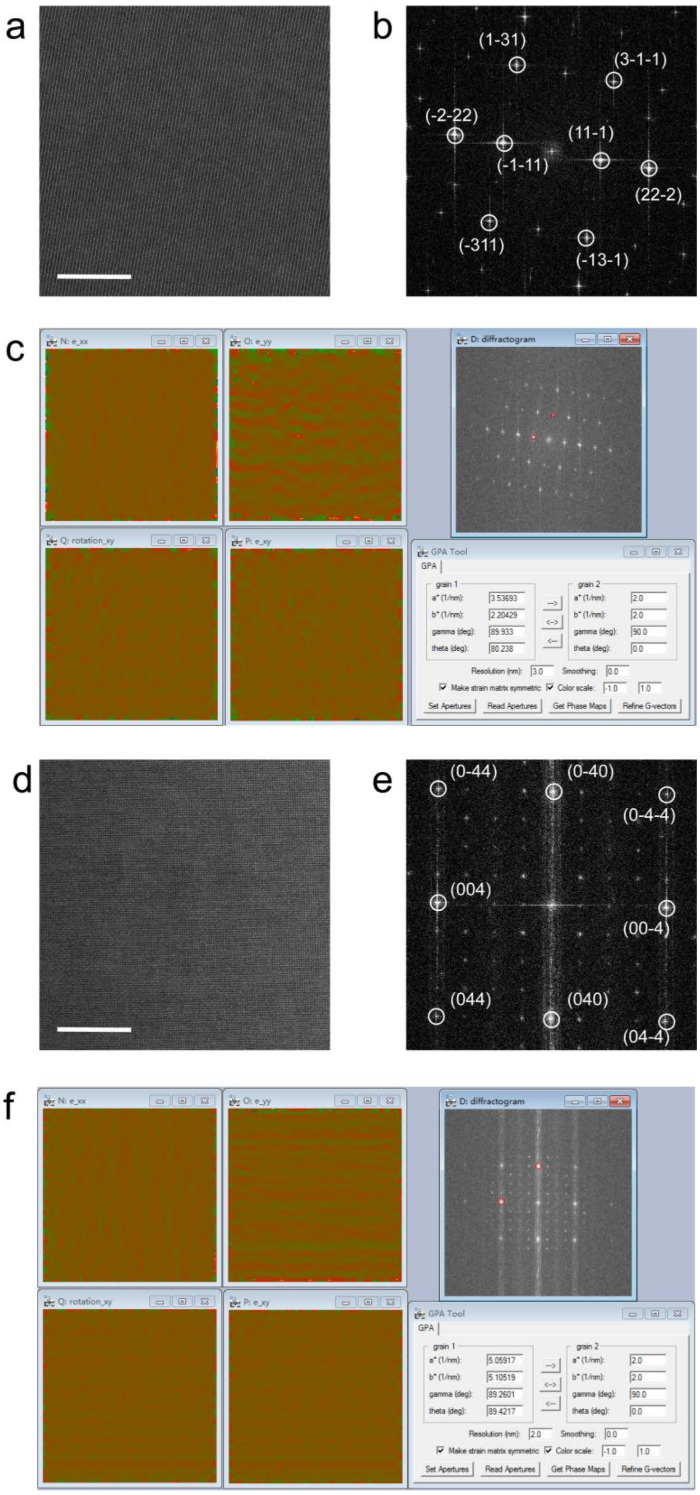

Supplementary Figure 2. Low magnification HAADF-STEM images of pristine LNMO sample with corresponding FFT pattern and GPA analysis, indicating no FIB damage to the sample. (a, b, c) show the results from [112] zone axis. (d, e, f) show results from [100] zone axis. Scale bar 10nm.

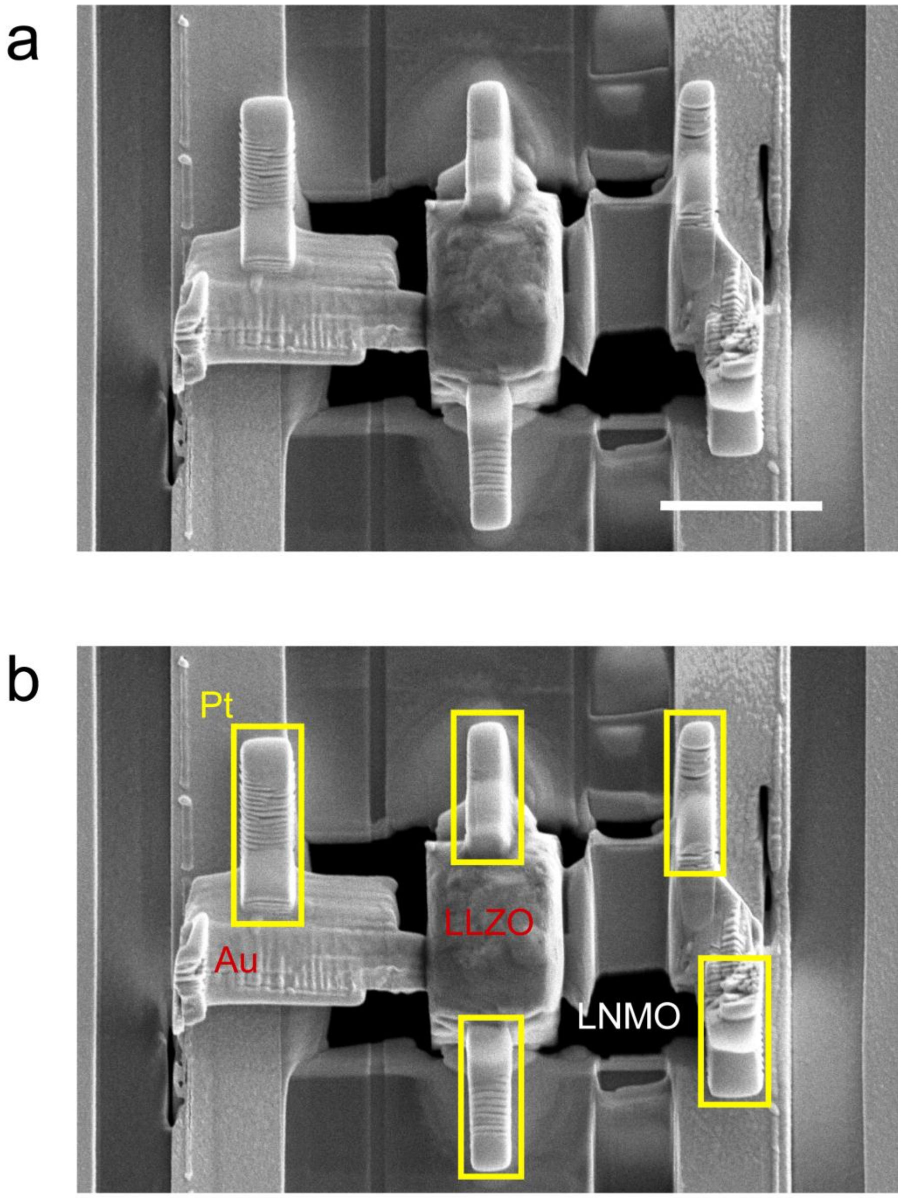

Supplementary Figure 3. SEM image illustrating the configuration of an assembled full-cell on the in situ chip. (a) SEM image of assembled full-cell. (b) Marked SEM image illustrating the full-cell configuration. Scale bar 5μm.

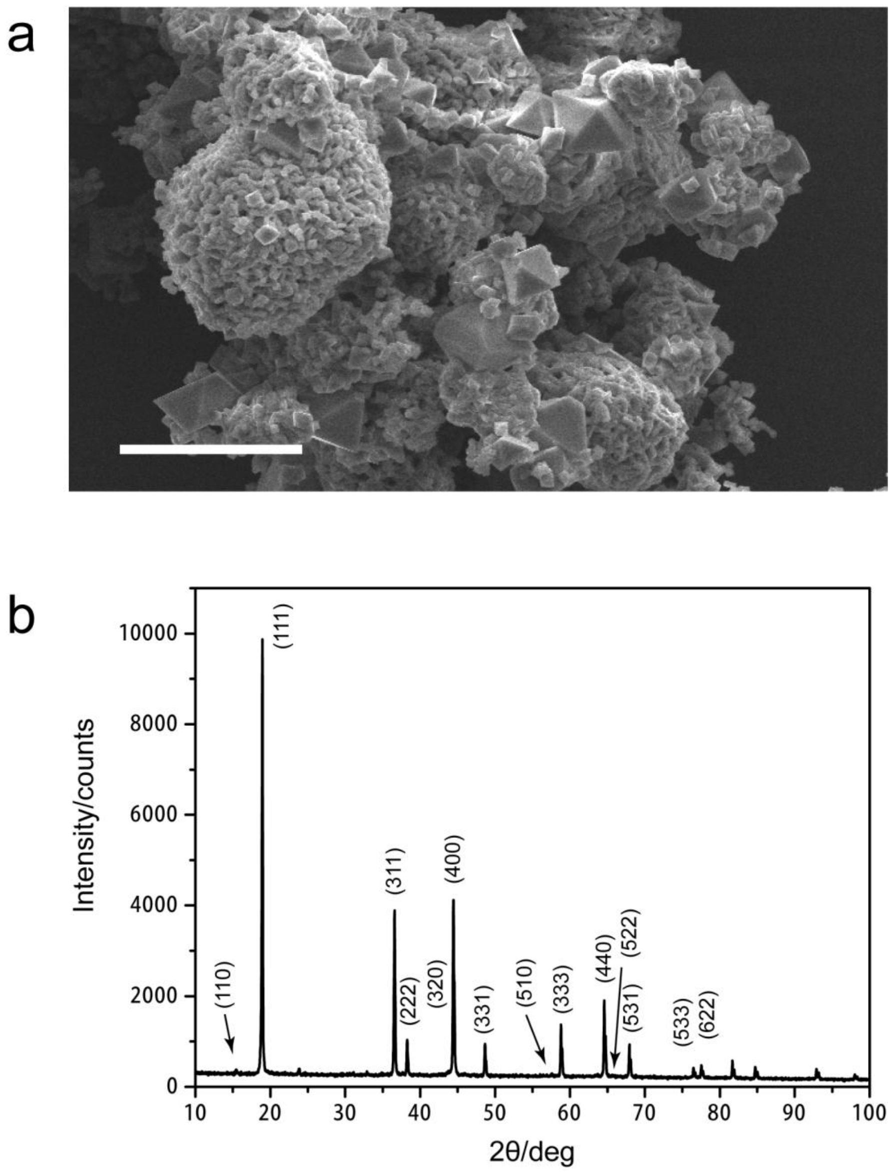

Supplementary Figure 4. SEM image and XRD spectrum of LNMO cathode material before FIB milling. (a) Octahedral morphology of the pristine single crystal LNMO particles with almost 3~10 micro-meters sizes. (b) XRD spectrum of the pristine LNMO, indicating its spinel $P4_332$ structure. Scale bar 10μm.

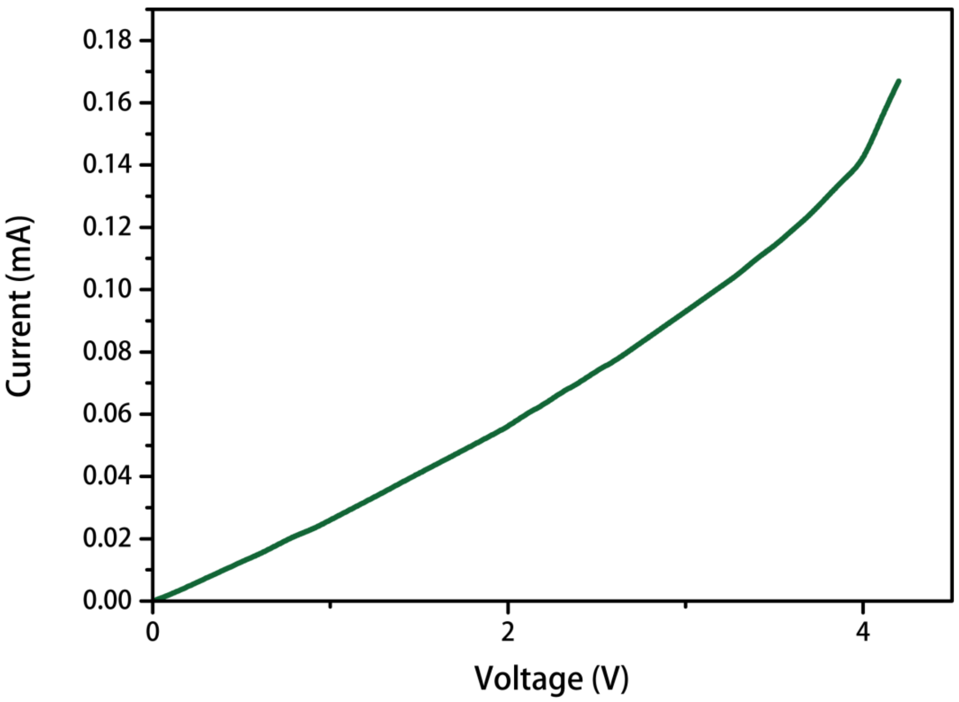

Supplementary Figure 5. Voltage-current plot of the in situ delithiation process. In this study, we manually tune the voltage step by step with 0.1V increase each step. It takes 30 to more than 60 seconds before increasing the voltage to next step to make sure an equivalent delithiation process. The charging process ended at 4.2V due to the broken of the LNMO cathode.

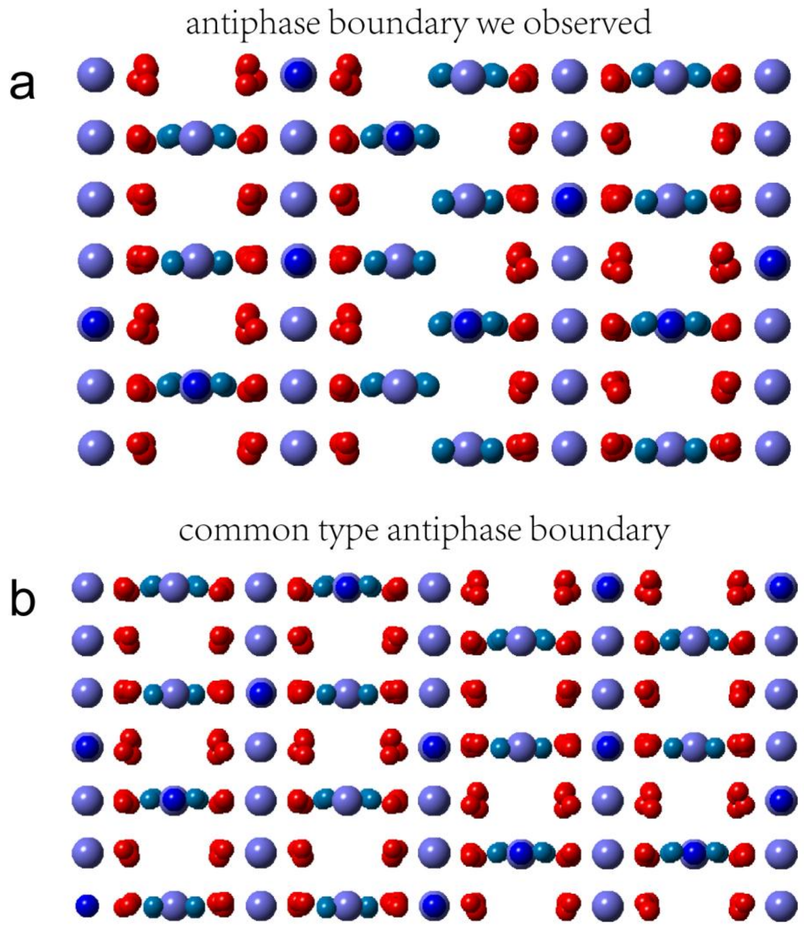

Supplementary Figure 6. Atomic models of antiphase boundaries. Atomic structure model of (a) antiphase boundary with one transition metal rich plane missing which we observed using in situ STEM and the common type antiphase boundary (b).

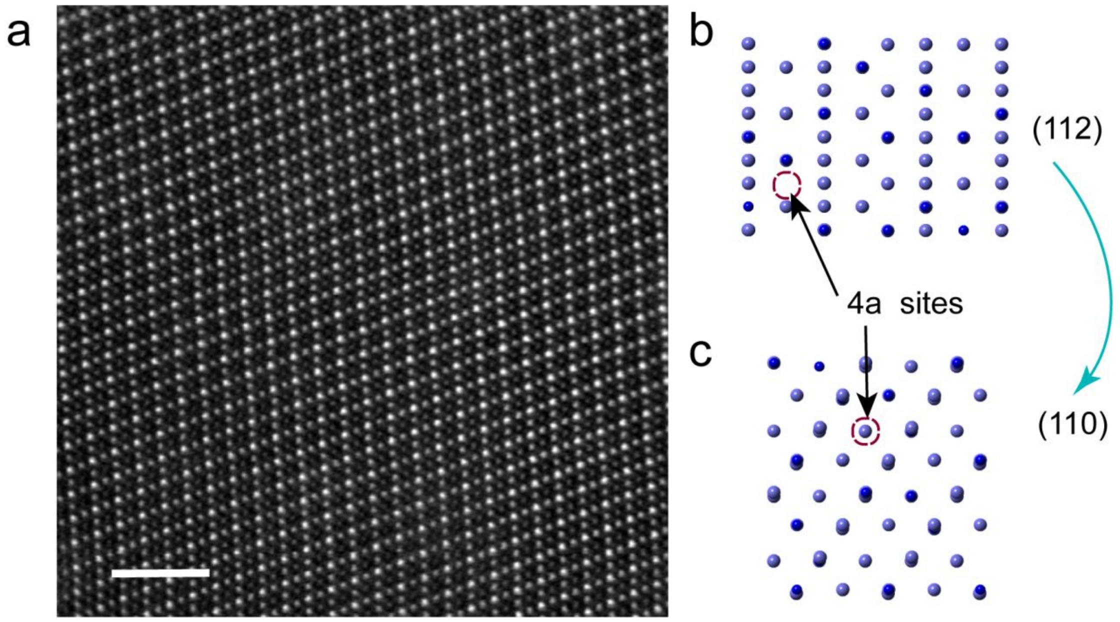

Supplementary Figure 7. In-situ atomic resolution HAADF-STEM results of <110> zone axes after delithiation. (a) In situ HAADF-STEM image of delithiated LNMO of <110> zone axes. (b) Antiphase boundary structure of <112> zone axes. (c) Structure of the model in (b) rotates to <110> zone axes, which is in consistence with the results in (a). Scale bar 2nm.

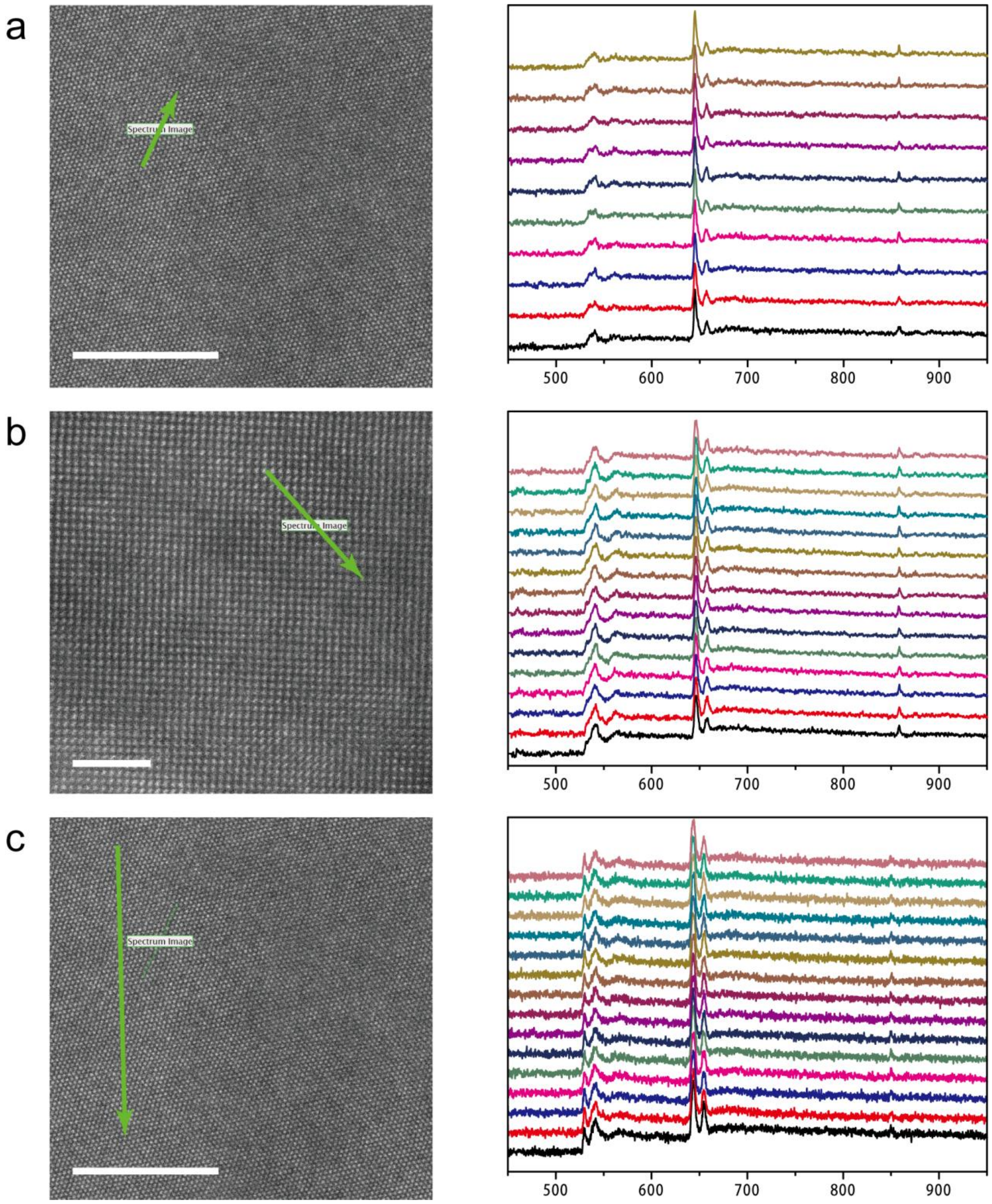

Supplementary Figure 8. STEM-EELS core-loss results. (a), (b) and (c) are core-loss EELS spectra of <111>, <100> and <110> zone axes after delithiation. Green arrows show the position and direction of EELS line scanning. Both HAADF image and its EELS high loss results show homogeneous property of atomic structure and electronic structure of <111>, <100> and <110> zone axes. Scale bar (a) 5nm, (b) 2nm, (c) 5nm.

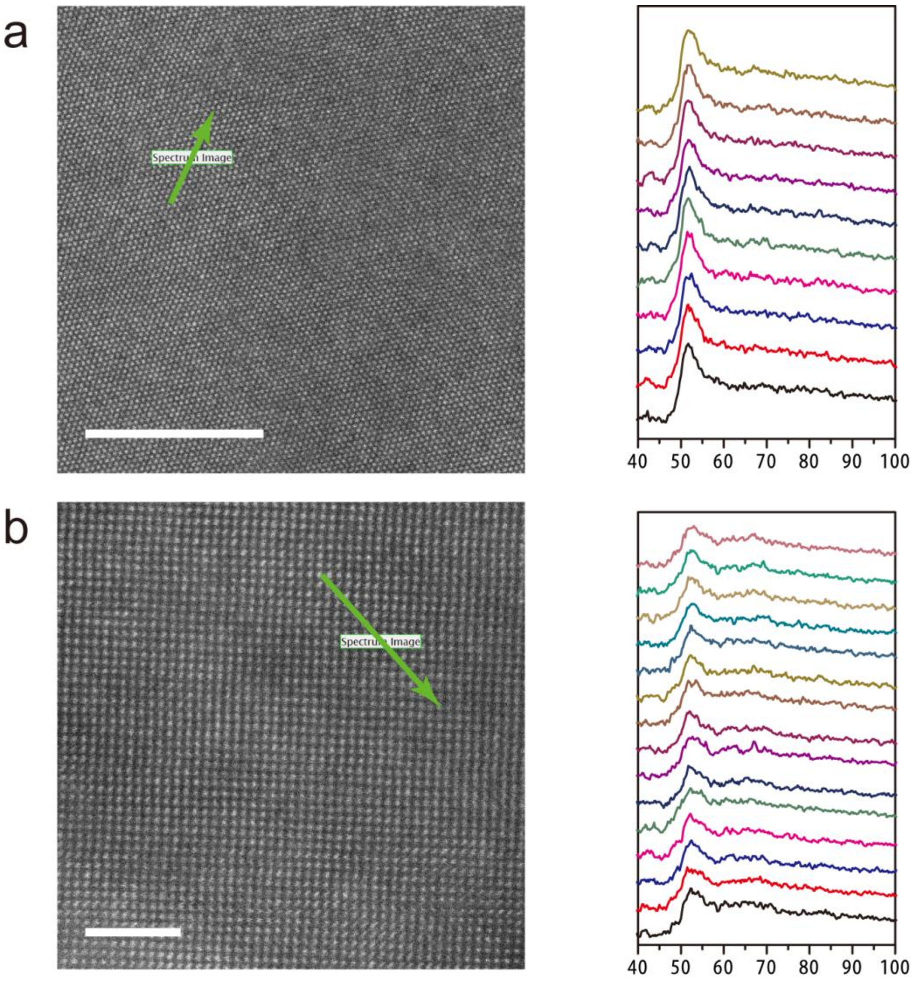

Supplementary Figure 9. STEM-EELS low-loss results. (a), (b) are low-loss EELS spectra of <111>, <100> zone axes after delithiation. Green arrows show the position and direction of EELS line scanning. Results show the overlap and weak signal of Mn, Li and Ni after delithiation, which lacks enough signal to noise ratio to analyze Li signal.[1] Scale bar (a) 5nm, (b) 2nm.

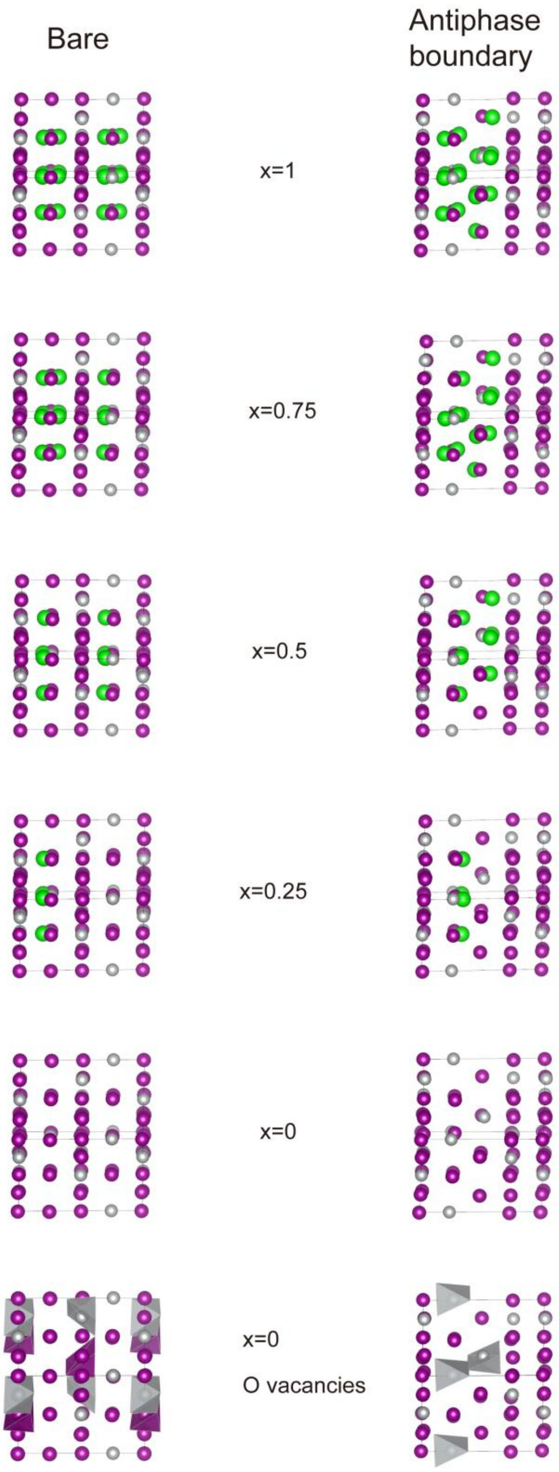

Supplementary Figure 10. Structure model used for theoretical calculations in Fig. 5a. Right and left columns are the intact LNMO structure and the antiphase boundary structure, respectively. X in middle column represents x in $Li_xNi_{0.5}Mn_{1.5}O_4$.

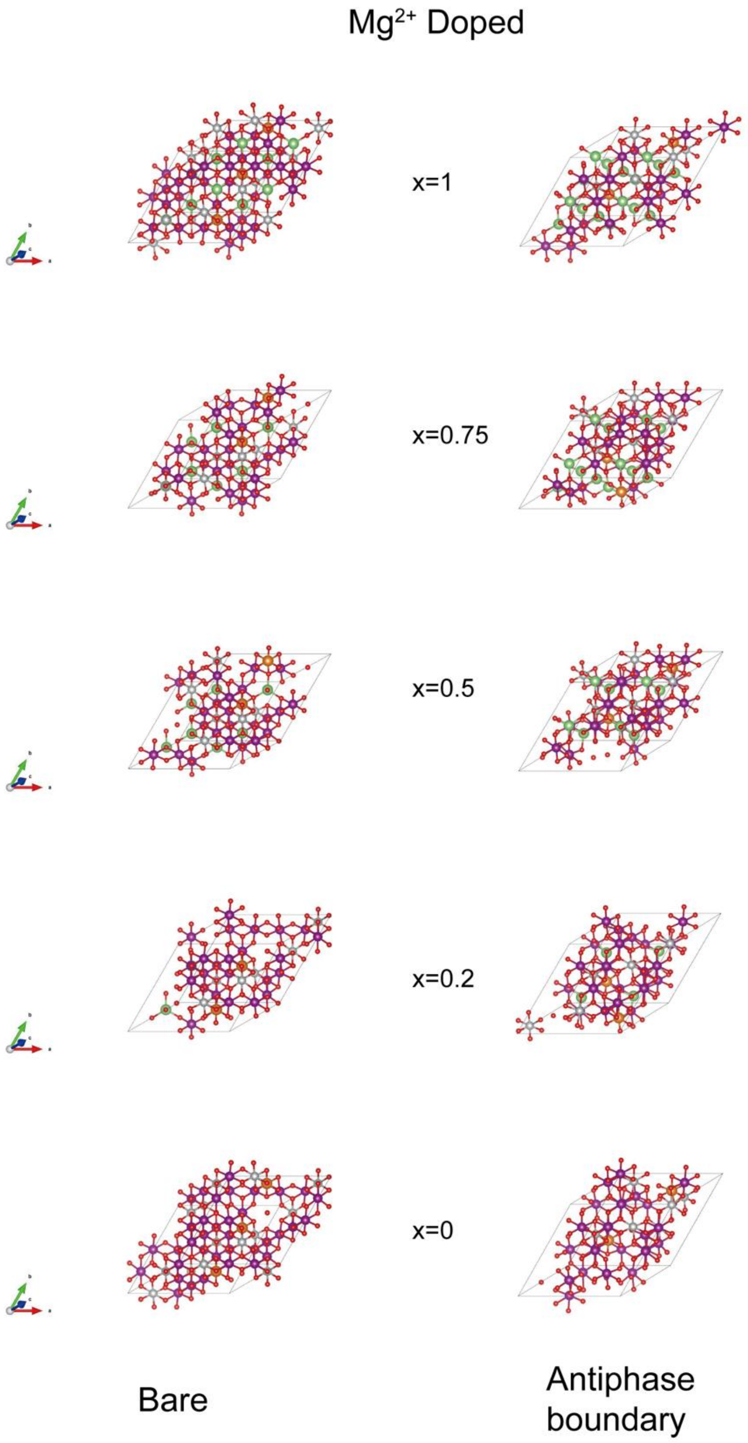

Supplementary Figure 11. Structure model used for theoretical calculations. Right and left columns are the doped structure with and without antiphase boundary, respectively. X in middle column represents x in $Li_{16x}Ni_6M_2Mn_{24}O_{64}$.

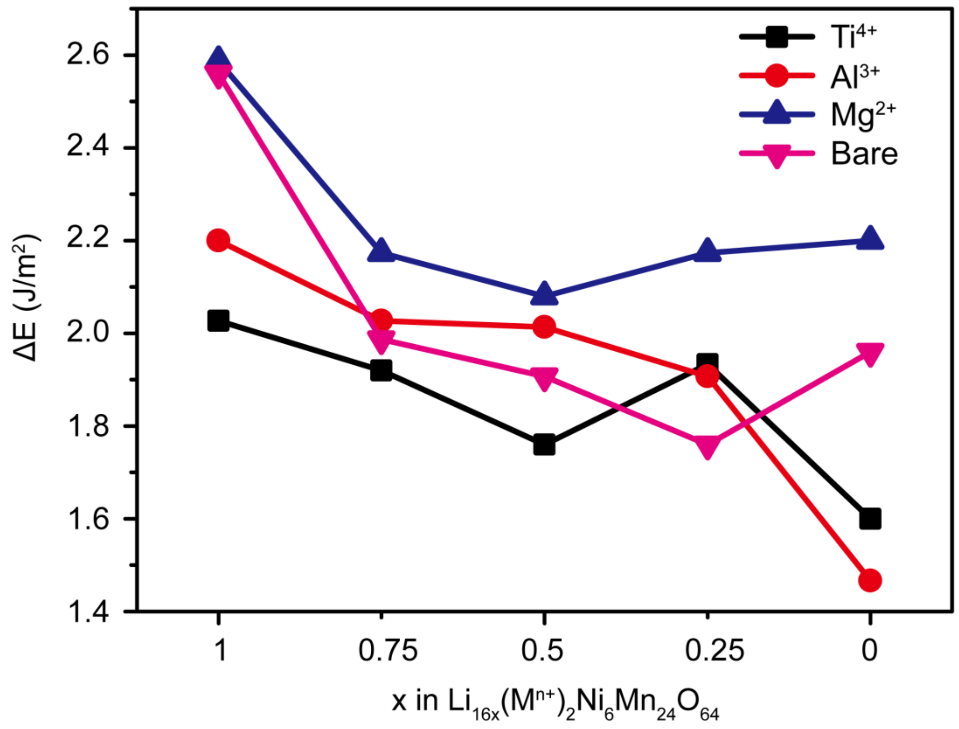

Supplementary Figure 12. Theoretical calculation results of the interface energy of different valence doping states. The pink line shows the result of the bare structure. The black, red, and blue colors represent Ti^{4+}, Al^{3+}, and Mg^{2+} doping results, respectively.

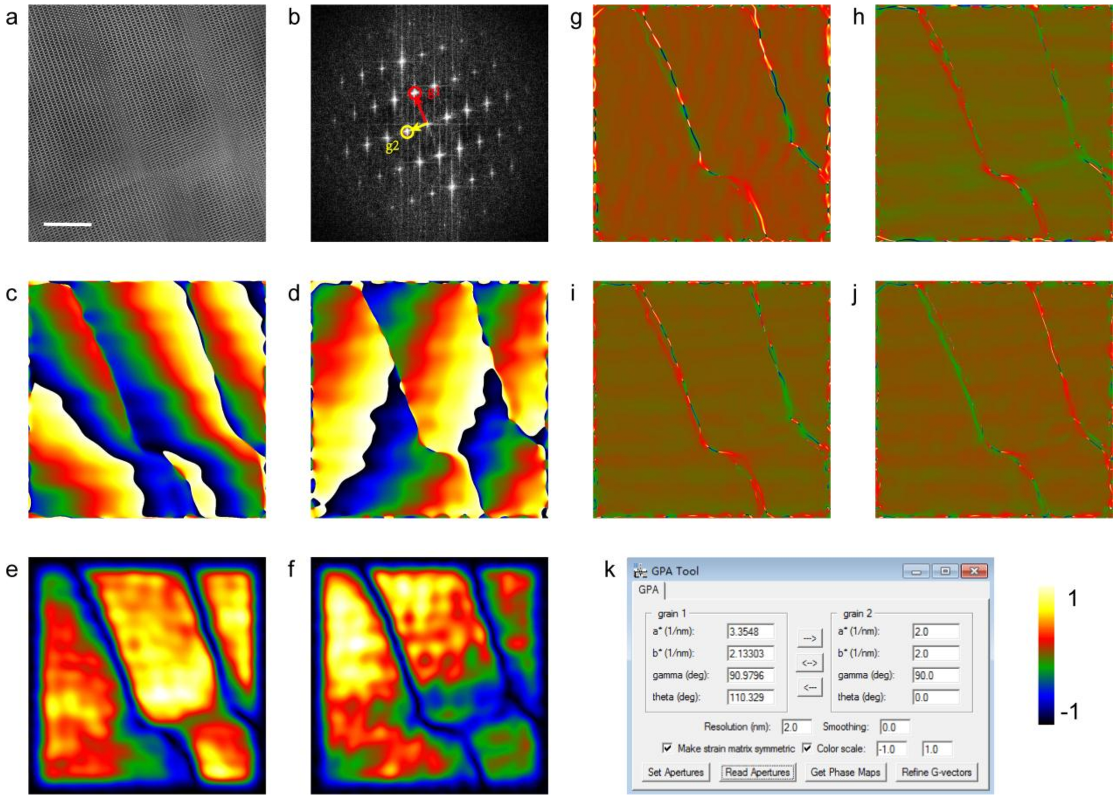

Supplementary Figure 13. Geometric phase analysis. (a) HAADF-STEM image in Fig. 2g. (b) FFT pattern of (a) with arrows and circles indicating selected g1 and g2. (c d) The geometric phase images calculated from g1 and g2 in (b). (e, f) are the scale of the reciprocal lattice vectors g1 and g2 respectively. (g-j) are the strain maps: ε_{xx}, ε_{yy}, ε_{xy}, and rotation respectively. (k) is the screen capture of the control panel in the GPA plugin. See details in Methods section of article and references[2, 3, 4]. Scale bar 5nm.

Supplementary Table

Supplementary Table 1. Data of calculation results of Figure 5 is listed. First and second columns list the data of Figure 5a, which indicates the energy difference of intact structure and structure with TM ions migration. Third to fifth columns list the data of Figure 5b and S11, indicating the energy difference of Ti^{4+}, Al^{3+} and Mg^{2+} cations doped structure separately.

Bare ($J\ m^{-2}$)	With O Vacancies ($J\ m^{-2}$)	Ti^{4+} ($J\ m^{-2}$)	Al^{3+} ($J\ m^{-2}$)	Mg^{2+} ($J\ m^{-2}$)
2.56	/	2.03	2.20	2.59
1.99	/	1.92	2.03	2.17
1.91	/	1.76	2.01	2.08
1.76	1.76	1.93	1.91	2.17
1.96	1.49	1.60	1.47	2.20

Supplementary Methods

Synthesis process of $P4_332$ LNMO

The $P4_332$ LNMO sample was obtained by using a ceramic route. Stoichiometric amounts of lithium (AR, 99%, Alfa), manganese ($Mn(NO_3)_2 \cdot 4H_2O$, AR, 98%, Alfa) and nickel ($Ni(NO_3)_2 \cdot 6H_2O$, AR, 98%, Alfa) nitrates were dissolved in ethanol. After evaporation of the solvent under continuous stirring at 80℃, the resulting paste was preheated at 500℃ for 2 h, and further annealed at 800℃ for 8 h. The sample was cooled naturally in the furnace which was switched off (cooling rate of about 5℃ /min). We further Annealed the obtained sample at 600℃ for 4 days in air, which was followed by natural cooling, leading to the formation of the $P4_332$ structure LMNO.

LLZO solid electrolyte

Y and Ta doped LLZO ($Li_{6.75}La_{2.84}Y_{0.16}Zr_{1.75}Ta_{0.25}O_{12}$) solid electrolyte is a product of Tai'an Faraday Energy Technology co. Ltd. The SEM image and corresponding XRD spectrum of LLZO solid electrolyte can be found in literature *JACS 2017, 139. 4274-4277*.

Au anode

Au anode is lifted out from Au wire with 0.3mm diameter and 99.99% purity. Au wire is the commercial product of Beijing Zhongxingbairui Technology Co., Ltd.

Supplementary References

1. Komatsu, H. et al. Solid solution domains at phase transition front of $Li_xNi_{0.5}Mn_{1.5}O_4$. *Adv. Energy Mater.* **5,** 1500638 (2015).

2. Hytch, M.J., Snoeck, E. & Kilaas, R. Quantitative measurement of displacement and strain fields from HREM micrographs. *Ultramicroscopy* **74,** 131–146 (1998).

3. Han, Y. et al. Sub-nanometre channels embedded in two-dimensional materials. *Nat. Mater.* **17,** 129–133 (2018).

4. Zhou, W. et al. Dislocation-driven growth of two-dimensional lateral quantum-well superlattices. *Sci. Adv.* **4,** eaap9096 (2018).
